# The tyrosine capsid mutations on retrograde adeno-associated virus accelerates gene transduction efficiency

**DOI:** 10.1186/s13041-022-00957-0

**Published:** 2022-08-08

**Authors:** Ryota Nakahama, Aika Saito, Sensho Nobe, Kazuya Togashi, Ikuo K. Suzuki, Akira Uematsu, Kazuo Emoto

**Affiliations:** 1 Department of Biological Sciences, Graduate School of Science, The University of Tokyo, 7-3-1 Hongo, Bunkyo-ku, Tokyo, 113-0033 Japan; 2 International Research Center for Neurointelligence (WPI-IRCN), The University of Tokyo, 7-3-1 Hongo, Bunkyo-ku, Tokyo, 113-0033 Japan

**Keywords:** Adeno-associated virus, Retrograde transport, Monosynaptic anterograde transport, Cortex, Limbic area

## Abstract

Adeno-associated virus (AAV) vector is a critical tool for gene delivery through its durable transgene expression and safety profile. Among many serotypes, AAV2-retro is typically utilized for dissecting neural circuits with its retrograde functionality. However, this vector requires a relatively long-term incubation period (over 2 weeks) to obtain enough gene expression levels presumably due to low efficiency in gene transduction. Here, we aimed to enhance transgene expression efficiency of AAV2-retro vectors by substituting multiple tyrosine residues with phenylalanines (YF mutations) in the virus capsid, which is previously reported to improve the transduction efficiency of AAV2-infected cells by evading host cell responses. We found that AAV2-retro with YF mutations (AAV2-retroYF)-mediated transgene expression was significantly enhanced in the primary culture of murine cortical neurons at 1 week after application, comparable to that of the conventional AAV2-retro at 2 week after application. Moreover, transgene expressions in the retrogradely labeled neurons mediated by AAV2-retroYF were significantly increased both in the cortico-cortical circuits and in the subcortical circuits in vivo, while the retrograde functionality of AAV2-retroYF was equally effective as that of AAV2-retro. Our data indicate that YF mutations boost AAV2-retro-mediated retrograde gene transduction in vivo and suggest that the AAV2-retroYF should be useful for efficient targeting of the projection-defined neurons, which is suited to applications for dissecting neural circuits during development as well as future clinical applications.

**Supplementary Information:**

The online version contains supplementary material available at 10.1186/s13041-022-00957-0.

## Introduction

A fundamental goal of neuroscience is to understand the structure of the brain and to decipher how the brain gives rise to functions such as perception, cognition and behavior in health and disease. One barrier to understand the brain is its complexity, in which millions to billions of neurons interconnect one another to organize neural circuits in the mammalian brain. To untangle the complicated networks, recombinant viral vectors have emerged as powerful tools for analyzing the circuit structure and function by expressing genes of interest in a target-specific manner.

Recombinant adeno-associated viruses (AAVs) are the most prominent virus vectors, as they mediate high-level transgene expression and evoke low cytotoxicity [1]. Transportation property is another important feature of AAV vectors in the context of the neuroscience research [2], which is typically composed of two properties; axonal and trans-synaptic transport. These properties differ among AAV serotypes. Most AAVs express the gene products at the cell body and dendrites in the injection site, which propagate to the long-range axonal projections in an anterograde manner [3, 3] In addition, some AAV serotypes exhibit retrograde spread from their uptake at axon terminals in the injection site [5]. Based on the retrograde property, AAV2-retro has been designed to mediate efficient retrograde access [5]. For trans-synaptic transport, a recent study clearly proved that AAV1 and AAV9 exhibit anterograde monosynaptic transport under certain conditions [6]. These AAV vectors are now recognized as powerful tools for dissecting neural circuits. However, AAV-mediated gene transduction is relatively slow compared to other virus vectors. For example, AAV2-retro requires at least 2 week-incubation period for sufficient transgene expression [7, 8], which often hampers dissection of neural circuits in the infant and adolescence stages.

To date, many gene therapy researches have attempted to enhance gene transduction efficiency of AAVs [9, 10]. It has been reported that missense mutations on the AAV capsid remarkably enhanced transgene expression efficiency [11, 12]. During AAV infection, surface-exposed tyrosine residues of AAV capsid are phosphorylated by EGFR-PTK, which likely causes AAV gene transduction to become less effective [13], in part due to ubiquitination-proteasome-mediated degradation [14]. The replacement of surface-exposed tyrosines of AAV capsid with phenylalanine (YF mutations) can avoid the phosphorylation by EGFR-PTK, which in turn increases the transgene expression efficiency in Hela cells and murine hepatocytes [11]. Indeed, multiple YF mutations on the surface-exposed tyrosine residues can robustly increase the transgene expression efficiency in murine embryo fibroblasts [15], Hela cells, murine hepatocytes [15, 16], and mouse retina [17, 18]. This facilitative effect of the multiple YF capsid mutations is consistently observed in multiple serotypes of AAVs [19–21]. However, it remains unresolved whether the YF mutations could affect retrograde and/or anterograde trans-synaptic AAV transgene expression.

In this study, we aimed to characterize whether the YF mutations could improve transgene expression efficiency of AAV2-retro in vitro and in vivo. We found that AAV2-retroYF significantly improved the transgene expression efficiency at 1 week after in vitro application. We further showed that the AAV2-retroYF increased transgene expression efficiency in the multiple neural circuits of the mouse brain such as the cortico-cortical and the subcortical circuits. By contrast, the YF mutations on AAV1 rather decreased the transsynaptic-anterograde transfer efficiency compared to the conventional AAV1. These data suggest that the YF mutations improve transgene expression efficiency in a serotype-dependent manner and that the AAV2-retro with YF mutations might accelerate functional dissection of the brain circuit in vivo.

## Materials and methods

All experiments were conducted in accordance with The University of Tokyo's Regulations for Animal Experiments and The University of Tokyo's Regulations for the Use of Genetically Modified Organisms.

### Animals

For in vitro experiments, we used pregnant ICR mice (Japan SLC Inc.) to extract embryonic cortical neurons. For in vivo AAV experiments, we used male adult C57BL/6 N (Japan SLC Inc.) and Ai14 (Cre-dependent tdTomato reporter) mice. For generation of Ai14 mice, the male homozygous Ai14 mice (Jackson Laboratories, RRID: IMSR_JAX:007,914) were crossed with C57BL/6 N (Japan SLC Inc.). The mice were kept in temperature- and humidity-controlled rooms with constant feeding and watering and a 12 h light/dark cycle.

### Plasmid construction

**Fig. 1:**
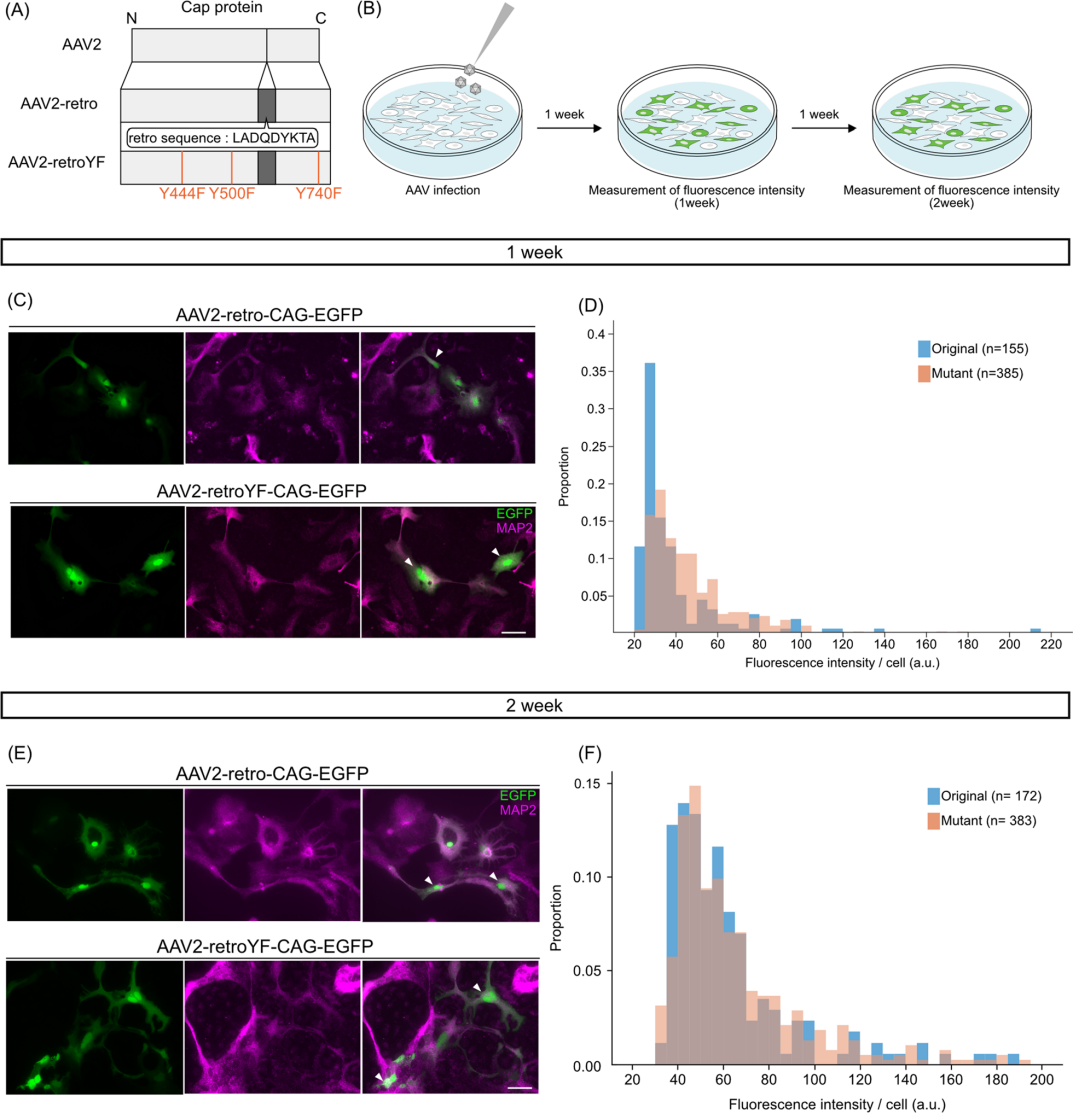
Rapid enhancement of transgene expressions in primary cultured cortical neurons by AAV2-retro YF mutation. **A** A schematic of the YF mutation sites of AAV2-retroYF. Note that the Y730F in AAV2YF shifts Y740F due to the 10 amino acids of the retro sequence insertion. **B** A schematic view of the experimental procedure of in vitro AAV assay on the primary cultured neuron. **C** Representative images of EGFP (green) and MAP2 (magenta) in AAV2-retro-CAG-EGFP (top) vs AAV2-retroYF-CAG-EGFP (bottom) applied cell cultures at 1 week post infection. The white arrows indicate EGFP- and MAP2-double positive neurons. **D** The histogram of the EGFP fluorescent intensity of the double positive neurons (n = 155 [original], n = 385 [mutant], p < 0.001, U = 18,296, Wilcoxon's rank sum test). **E** Representative images of EGFP (green) and MAP2 (magenta) in AAV2-retro-CAG-EGFP (top) vs AAV2-retroYF-CAG-EGFP (bottom) applied cell cultures at 2 week post infection. The white arrows indicate EGFP and MAP2 double positive neurons. **F** The histogram of the EGFP fluorescent intensity of double positive neurons at 2 week post infection (n = 172 [original], n = 383 [mutant], p = 0.11, U = 30,146, Wilcoxon's rank sum test). All scale bar, 50 μm

A procedure based on QuikChange II Site-Directed Mutagenesis Kit (Agilent, Catalog#: 200523) was performed using rAAV2-retro helper (Addgene, #81070) and pRC1 Vector (Takara, Production code 6673, 6668 and 6669) to produce pAAV2-retroYF and pAAV1YF (Fig. 1A, Additional file 1: Fig. S2A). The mutation procedure was repeated multiple times and the mutation was inserted one by one. We introduced the mutations at the same site as a previous report[15].

### Viral constructs

AAV2-retro-CAG-EGFP and AAV2-retroYF-CAG-EGFP were packaged at IRCN Virus Core (The University of Tokyo, Japan). AAV1-hSyn-Cre was purchased from Addgene (105553-AAV1). AAV1YF-hSyn-Cre was packaged at and gifted from Johansen Lab (Center for Brain Science, RIKEN, Japan).

### Cell culture and imaging

AAV-293 cells were cultured and maintained in DMEM medium containing 10% FBS (GE Healthcare, Code: SH30396.03), 1% GlutaMAX™ Supplement (Thermo Fisher Scientific, Cat. 35050-061), 0.5% Penicillin–Streptomycin (Thermo Fisher Scientific, Cat. 15070-063) under 5% of CO2 at 37℃. After seeding, the virus (AAV2-retro-CAG-EGFP or AAV2-retroYF-CAG-EGFP (titers of 9.7 × 10^12^ vg/mL) was applied into the culture medium. Cells were imaged at 29 h post infection. The fluorescence images were obtained using a fluorescence microscope (LEICA DMI6000B) with a 10 × objective. Then the fluorescence intensity of the cells was measured with a software (Image J).

The primary cultured neurons were prepared from the cerebral cortex of ICR mouse embryos at E17. The cerebral cortices were washed with cold HBSS (SIGMA-ALDRICH, Cat. H6648) and then digested with DMEM supplemented with 0.05%Trypsin–EDTA (Wako, Cat.209-16941) for 20 min at 37 °C. The tissues were then triturated with a pipet. The dissociated cells were pelleted, resuspended, and plated with DMEM/F-12 Ham’s growth medium (SIGMA-ALDRICH, Cat. D8062) containing 10% FBS (GEHealthcare, Code:SH30396.03),1% GlutaMAX™ Supplement (Thermo Fisher Scientific, Cat. 35050-061), 0.5% Penicillin–Streptomycin (Thermo Fisher Scientific, Cat. 15070-063). For immunostaining, the dissociated cortical neurons were grown on the glass coverslips (Corning) pre-coated with poly-d-lysine (PDL). After the seeding, the virus (AAV2-retro-CAG-EGFP or AAV2-retroYF-CAG-EGFP; titers of 9.7 × 10^12^ vg/mL) was applied into the culture medium at the concentration of MOI 5000 (5000 virus particles per cell). Cells were fixed with 4% PFA at 1- or 2-week post infection and immunostained with mouse anti-MAP2 monoclonal antibody in order to define neurons. The fluorescence images were obtained using a fluorescence microscope (BZ-X810, KEYENCE) with a 40 × objective lens. Then the fluorescence intensity of the cells was automatically measured with a software (KEYENCE Image Analyzer). During the whole imaging and analysis process, the information of samples (the original virus or the mutant virus) was blinded to the experimenter.

### Virus injection

All surgeries were performed under aseptic conditions as described in elsewhere [22]. Mice were anesthetized with isoflurane (3% for induction, 1.5% for maintenance). We injected 300 nL of the virus solution (10 nL/s) at the following coordinates (S1: AP 0.0 mm, ML 2.0 mm, DV − 0.5 mm; lateral hypothalamic area (LHA): AP − 1.0 mm, ML 1.2 mm, DV − 4.9 mm; V1: AP − 3.9 mm, ML 2.6 mm, DV − 0.5 mm). For the transgene expression efficiency experiments of retrograde viruses, AAV2-retro-CAG-EGFP or AAV2-retroYF-CAG-EGFP (titers of 9.7 × 10^12^ vg/mL) was injected at the S1 or LHA. For the retrograde transport efficiency experiments, AAV2-retro-CAG-EGFP or AAV2-retroYF-CAG-EGFP was mixed with the same amount of Red Retrobeads IX (Lumafluor). For the anterograde trans-synaptic virus experiments, we injected AAV1-hSyn-Cre or AAV1YF-hSyn-Cre at the V1 (titers of 9.7 × 10^12^ vg/mL).

### Brain sectioning and imaging

The mice were transcardially perfused with phosphate-buffered saline (PBS) followed by 4% PFA-PBS solution. The brains were post-fixed in 4% PFA-PBS for overnight at 4 °C and placed in 30% sucrose PBS solution for 2 days. Coronal sections were obtained using a cryostat with a thickness of 40 μm. The sections were analyzed using a fluorescence microscope (KEYENCE BZ-X810) with a 10X, 20X or 40X objective. For AAV2-retro experiments, the sections including the primary cortical sensory area (S1) or the nucleus accumbens (NAc) were imaged. The fluorescence intensity of the cells and the number of cells were automatically measured with a software (KEYENCE Image Analyzer). Since retrograde projections are sparse in the above-mentioned circuits, we quantified GFP signal to evaluate the transduction efficiency as conducted in the other studies [23–25]. For AAV1 experiments, fifty consecutive sections including the superior colliculus (SC) were imaged per a mouse. The number of cells was automatically measured with a software (KEYENCE Image Analyzer). During the whole imaging and analysis processing, the information of samples (the original virus or the mutant virus) was blinded to the experimenter.

### Statical analysis

For comparison of the fluorescence intensities between the original virus and the mutant virus, histograms were drawn for all cells using MATLAB R2019b. We performed Lillifors tests and the null hypothesis was rejected at the 0.1% level of significance so the data was not considered to be derived from a normal distribution. Therefore, we performed a Wilcoxn's rank sum test to assess the effect of the YF mutations on fluorescence intensity.

For comparison of the number of cells between the original virus and the mutant virus, the ratio of the number of cells positive for both EGFP and Red Retrobeads to the number of Retrobeads-positive cells was calculated as the index of the retrograde transfer efficiency in AAV2-retro experiments. The number of tdTomato positive cells were counted as the index of the anterograde transfer efficiency in AAV1 experiments. Then an unpaired two-tailed Welch's t-test was performed. These analyses were conducted with MATLAB2019b or Graphpad prism 8.0 software. Differences were considered statistically significant at p < 0.05 with a confidence limit of 95%.

## Results

### The YF mutations significantly accelerate AAV2-retro-mediated transgene expression in the primary cultured neurons

To evaluate the YF mutations on the expression efficiency of AAV2-retro in vitro, the original virus (AAV2-retro-CAG-EGFP) or the mutant virus (AAV2-retroYF-CAG-EGFP) was applied into the primary cortical neuron cultures from mice (Fig. 1A, B). We then measured the fluorescence intensity of EGFP per cell labeled by the neuronal marker MAP2. We found that the fluorescence intensity of EGFP was significantly increased in neurons infected with the YF mutant virus compared to the original virus at 1 week post infection (n = 155 [original], n = 385 [mutant], p < 0.001, U = 18,296, Wilcoxon's rank sum test; Fig. 1C, D). The enhancement of EGFP intensity by AAV2-retroYF was no longer detectable at 2 weeks post infection (n = 172 cells [original], n = 383 cells [mutant], p = 0.11, U = 30,146, Wilcoxon's rank sum test; Fig. 1E, F), suggesting that the YF mutations likely accelerate the gene expression speed, rather than the final gene expression levels. Notably, in contrast to the primary cultured neurons, we observed reduced EGFP expression by the AAV2-retroYF compared to the original virus in AAV-293 cells (Additional file 1: Fig. S1) (n = 75 cells [original], n = 75 cells [mutant], p < 0.001, U = 1315, Wilcoxon's rank sum test). It is thus likely that the YF mutations accelerate AAV2-retro-medicated transgene expression in the cultured primary neurons, but not in non-neuronal cells.

### The YF mutations on AAV2-retro significantly increased the transgene expression in the contralateral cortical projections

**Fig. 2:**
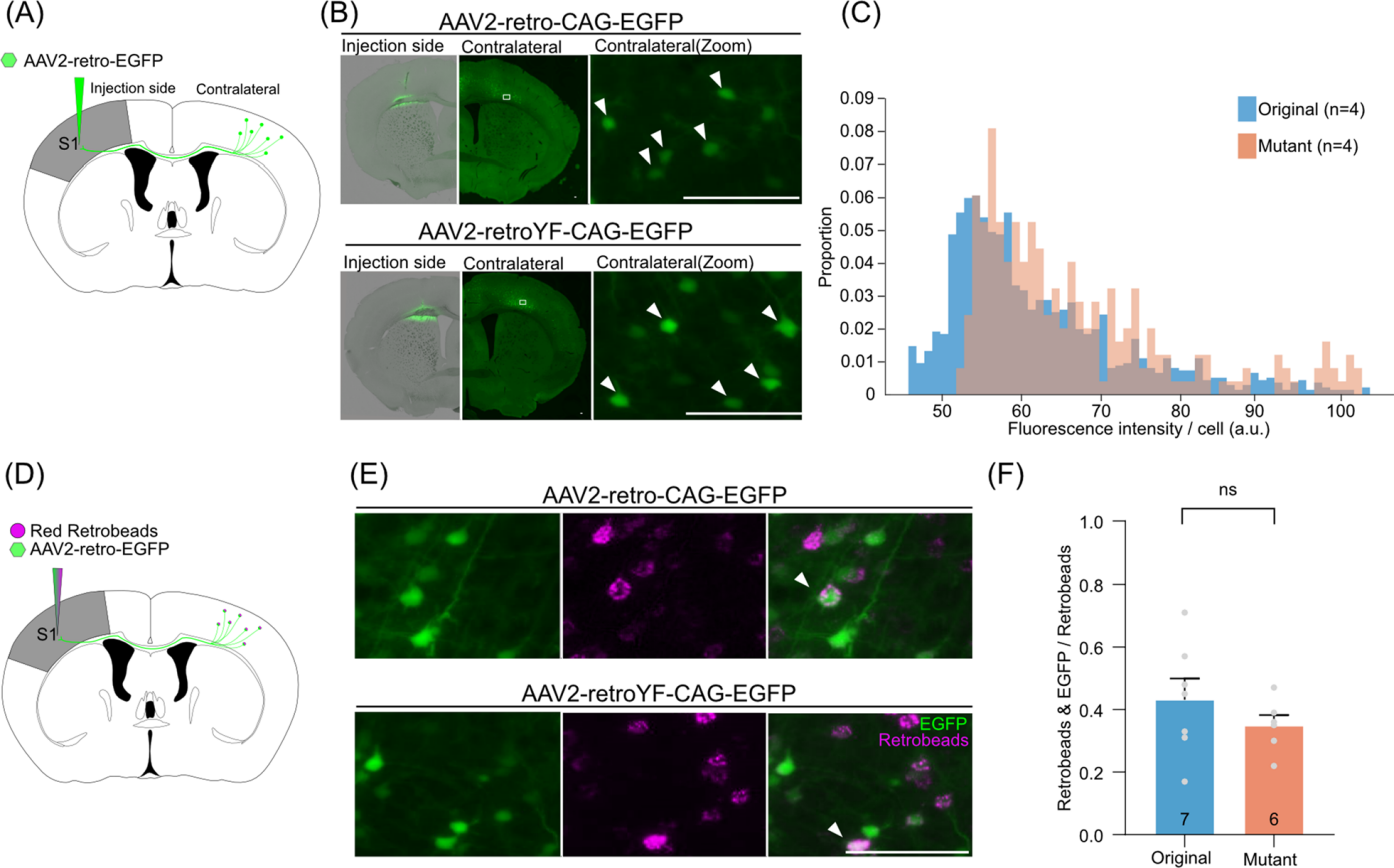
Increased gene transduction efficiency of AAV2-retro YF mutation in cortico-cortical projections. **A** A schematic of the expression efficiency assay in the cortico-cortical projection. The original or mutant virus was injected into the S1 cortex of the mouse brain. **B** Representative images of the injection and the contralateral sides in AAV2-retro-CAG-EGFP (top) and AAV2-retroYF-CAG-EGFP (bottom). EGFP signals were counted at the contralateral sides. The white arrows indicate EGFP labeled neurons in the contralateral sides (right). **C** The histogram of the fluorescent intensity of EGFP-positive neurons (n = 4 mice [original], n = 4 mice [mutant], p < 0.001, U = 123,049, Wilcoxon's rank sum test). **D** A schematic of the retrograde transfer efficiency assay. The original or mutant virus was mixed with Red Retrobeads and injected into the S1 cortex of the mouse brain. **E** Representative images showing EGFP signals (green) and retrobeads (magenta) in the contralateral S1 regions. The white arrows indicate EGFP- and retrobeads-double positive neuron. **F** The mean ratio of the number of retrobeads- and EGFP- positive neurons out of all retrobeads-positive neurons is shown (mean ± sem; n = 7 mice [original], n = 6 mice [mutant], p = 0.303, Welch's two-tailed t-test, t = 1.093, df = 8.789). All scale bars, 100 μm

Taking advantage of the rapid transgene expression of AAV2-retroYF in neuronal cultures, we next examined the transgene expression efficiency of AAV2-retro in vivo at 1 week post infection. The original virus or the mutant virus was injected into the cortical S1 region (Fig. 2A). Since the S1 neurons are known to project to the contralateral area through the corpus callosum, we measured the fluorescence intensity per cell in the contralateral S1 region, and found that the fluorescence intensity of EGFP was significantly increased in the cortical S1 region of the mutant virus-injected brain compared with that of the original virus (n = 4 mice [original], n = 4 mice [mutant], p < 0.001, U = 123,049, Wilcoxon's rank sum test; Fig. 2C).

We next tested if YF mutations might enhance the retrograde transfer efficiency of AAV2-retro or not. To this end, we injected the cocktail of virus (the original or the mutant) and the retrograde tracer (Red Retrobeads IX) simultaneously into the S1 region (Fig. 2D) and counted the number of cells with the fluorescence in the contralateral S1. The ratio of both Red Retrobeads IX- and EGFP-positive cells to Red Retrobeads IX-positive cells was not significantly different between the original virus and the mutant virus (n = 7 mice [original], n = 6 mice [mutant], p = 0.303, Welch's two-tailed t-test, t = 1.093, df = 8.789). These data suggest that the YF mutations increase the transgene expression efficiency of AAV2-retro in the cortical neurons without affecting transport efficiency.

### The YF mutations on AAV2-retro significantly increased the transgene expression in the NAc-LHA pathway

**Fig. 3:**
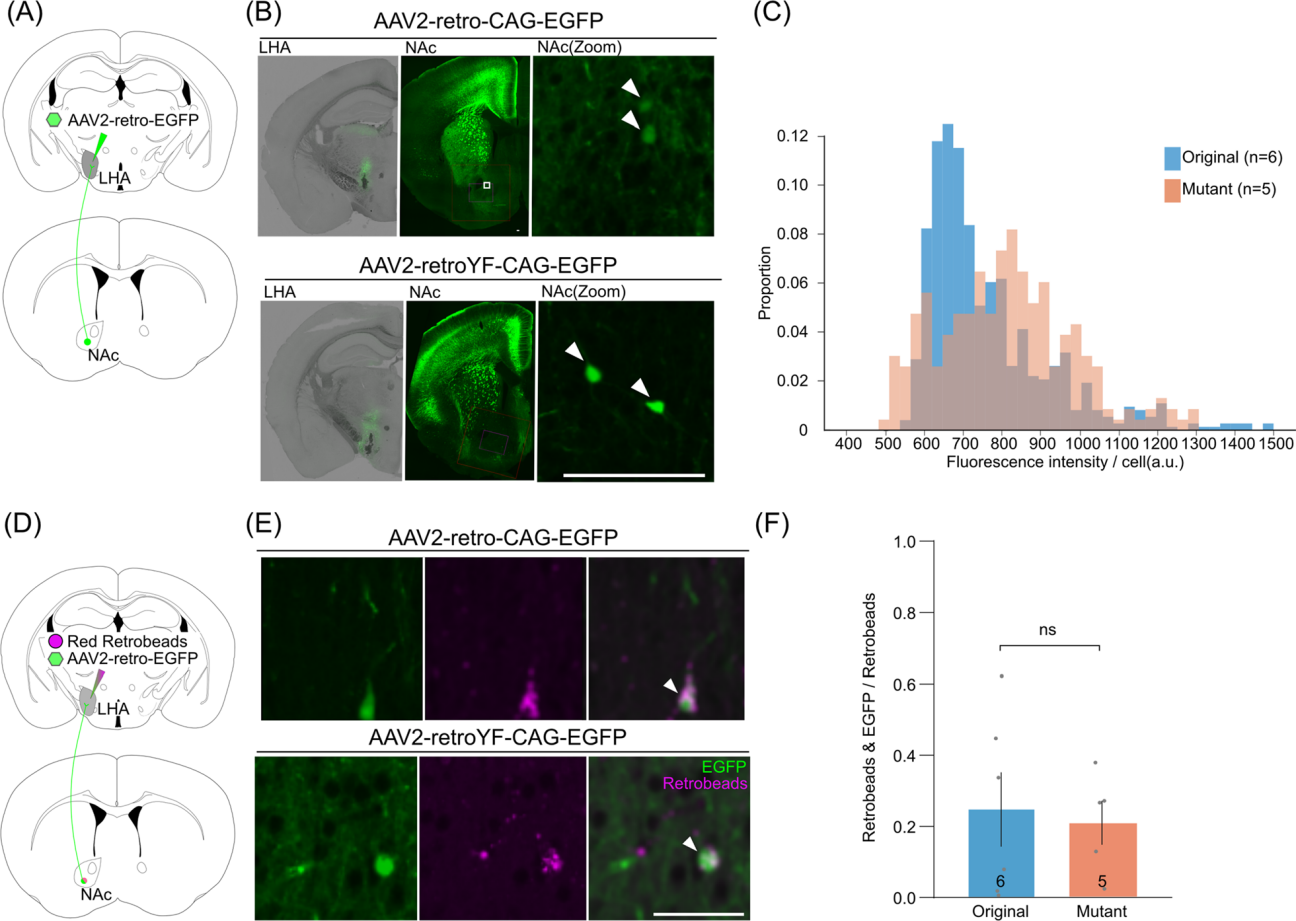
Increased gene transduction efficiency of AAV2-retro YF mutation in NAc-LHA pathway. **A** A schematic of the injection strategy targeting NAc-LHA projection. **B** Representative images of AAV2-retro-CAG-EGFP (top) and AAV2-retroYF-CAG-EGFP (bottom). The white arrows indicate EGFP-labeled neurons in the NAc. Scale bar, 100 μm. **C** The histogram of the fluorescent intensity of EGFP positive neurons in the NAc (n = 6 mice [original], n = 5 mice [mutant], p < 0.001, U = 45,325, Wilcoxon's rank sum test). **D** A schematic of retrograde transfer efficiency assay in the NAc-LHA circuits. The original or mutant virus was mixed with red retrobeads and injected into the LHA. **E** Representative images showing EGFP signals (green) and Red Retrobeads (magenta) in the contralateral S1. The white arrows indicate EGFP- and retrobeads-double positive neurons. Scale bar, 50 μm. **F** The mean ratio of the number of retrobeads- and EGFP-positive neurons out of all etrobeads IX-positive neurons (mean ± sem; n = 6 mice [original], n = 5 mice [mutant], p = 0.939, Welch's two-tailed t-test, t = 0.08176, df = 4.257)

Next, we assessed the effect of the YF mutations in AAV2-retro on the transgene expression efficiency and the transfer efficiency in a subcortical circuit (NAc-LHA), where original AAV2-retro has been reported to exhibit less than one-fifth efficiency compared to G-deleted rabies virus [26]. To evaluate the expression efficiency of transduced fluorescent protein in the NAc-LHA circuit, we injected AAV2-retro-EGFP viruses (original or mutant) in the LHA and analyzed EGFP fluorescent intensity in the NAc (Fig. 3A). We found that the fluorescence intensity of EGFP observed in the NAc neurons was significantly increased in the mutant virus compared with the original virus (n = 6 mice [original], n = 5 mice [mutant], p < 0.001, U = 45,325, Wilcoxon's rank sum test; Fig. 3B, C).

To investigate the effect of the YF mutations on the transfer efficiency in the NAc-LHA pathway, we injected the cocktail of AAV2-retro-EGFP viruses (original or mutant) and Red Retrobeads IX simultaneously into the LHA and counted the number of the EGFP/Red ratio in Red Retrobeads-positive neurons in the NAc (Fig. 3D). As a result, the EGFP/Red ratio in Red Retrobeads-positive neurons was not significantly different between the original virus and the mutant virus (n = 6 mice [original], n = 5 mice [mutant], p = 0.761, Welch's two-tailed t-test, t = 0.31532, df = 7.908; Fig. 3E, F). Thus, likewise the contralateral cortical pathway, YF mutations increase the transgene expression efficiency of AAV2-retro in the LHA neurons without affecting transport efficiency.

### The YF mutations on AAV1 reduced anterograde trans-synaptic efficiency

AAV1 is reported to exhibit an anterograde mono-synaptic transfer in a certain condition with minor retrograde transfer [6]. To examine whether the YF mutations could improve the anterograde monosynaptic transport, we introduced the same YF mutations into AAV1 vector. We then investigated the V1-SC pathway, which is known as the unidirectional neural circuit as shown previously [6]. We injected the original virus (AAV1-hSyn-Cre) or the mutant virus (AAV1YF-hSyn-Cre) into the V1 region of Ai14 mice that express tdTomato under the presence of Cre protein. We then evaluated the effect of the YF mutations by counting the number of tdTomato labeled neurons in the SC. We found that the number of cells labeled by tdTomato was significantly decreased in the mutant virus (n = 6 mice [original], n = 6 mice [mutant], p = 0.046, Welch's two-tailed t-test, t = 2.565, df = 5.384: Additional file 1: Fig. S2) , indicating that, unlike the AAV2-retro, the YF mutations reduce the anterograde transfer efficiency of AAV1.

## Discussion

In this study, we have shown that the YF mutations in the AAV2-retro vector significantly enhance the expression efficiency in the long-range projecting neurons. This notion is consistent with the previous studies that show facilitative effect of the YF mutations in the other AAV serotypes [19–21]. It is thus possible that the enhanced gene transduction in the AAV2-retroYF could follow the same mechanisms underlying other YF-mutated AAVs, in which the YF-mutated AAV is transported to the nucleus of infected cells more effectively by evading the sequential flow of the tyrosine phosphorylation, ubiquitination, and proteasome-mediated degradation, though we need further studies to test this mechanism.

The ratio of retrogradely labeled cells by AAV2-retroYF showed no significant difference both in the cortico-cortical and the NAc-LHA circuits (Figs. 2F, 3F), suggesting that the YF mutations in AAV2-retro do not seem to improve the retrograde functionality. The retrograde transfer efficiency of AAV2-retro could be potentially determined by multiple factors such as cellular uptake defined by the combination of the capsid surface ligand and the cell surface receptors. In line with this notion, it is reported that the modification of the receptor binding site of AAV9 improves the retrograde transfer efficiency, in which the mutated amino acids are different from the YF mutations [3]. To our knowledge, AAV2-retro receptors in neurons have not yet identified, while several studies have identified serotype-specific/non-specific AAV receptors [27–33]. AAV2-retro could fail to infect certain neurons which do not express endogenous AAV2-retro receptors. In this scenario, viral receptor complementation strategy would be a potential approach to overcome neurotropism, as has been developed in another retrograde virus [34]. Further studies will be needed to increase the retrograde transfer efficiency of AAV2-rero by focusing on AAV2-retro capsid surface ligands and neuron receptors.

In this study, we also introduced the YF mutations on AAV1 and found that the YF mutations rather reduce the efficiency of the mono-synaptic anterograde labeling (Additional file 1: Fig. S2). After the anterograde axonal transport, AAV1 is thought to infect postsynaptic neurons by being released through synaptic vesicles in a vesicle-associated membrane protein 2 dependent manner [35]. It is plausible that the YF mutations might disturb the distal axonal transport and/or endocytosis due to possible changes caused by the YF mutations such as conformational changes of the virus capsid or loss of target domains necessary for binding to synaptic vesicles.

In summary, we demonstrate that the YF mutations on the capsid of AAV2-retro significantly enhance the expression efficiency but not affect the retrograde transfer efficiency in multiple circuits of the mouse brain, whereas the YF mutations exhibited rather inhibitory effects on the anterograde mono-synaptic transportation of AAV1. With these findings, it is feasible to engineer the AAV vector tools that can permit a rapid retrograde access to specific projection neurons in the brain. Since YF mutated AAVs are applied to improve transgene expression in clinical trials, we speculate that AAV2-retroYF could be also useful for projection-specific gene manipulations in the future translational research.

## Supplementary Information

**Additional file 1: Fig. S1.** The gene transduction efficiency of AAV2-retro in AAV-293 cell was significantly decreased by YF mutations. **Fig. S2**. The number of neurons labeled by AAV1 was significantly decreased in the V1-SC pathway.

## Data Availability

Data will be made available upon reasonable request.

## Abbreviations

AAV: Adeno-associated virus
S1: Primary cortical sensory area
LHA: Lateral hypothalamic area
NAc: Nucleus accumbens
SC: Superior colliculus

## References

[1] Howard DB, Powers K, Wang Y, Harvey BK (2008). Tropism and toxicity of adeno-associated viral vector serotypes 1, 2, 5, 6, 7, 8, and 9 in rat neurons and glia in vitro. Virology.

[2] Saleeba C, Dempsey B, Le S, Goodchild A, McMullan S (2019). A student’s guide to neural circuit tracing. Front Neurosci.

[3] Castle MJ, Gershenson ZT, Giles AR, Holzbaur ELF, Wolfe JH (2014). Adeno-associated virus serotypes 1, 8, and 9 share conserved mechanisms for anterograde and retrograde axonal transport. Hum Gene Ther.

[4] Taymans J-M, Vandenberghe LH, Van Den Haute C, Thiry I, Deroose CM, Mortelmans L (2007). Comparative analysis of adeno-associated viral vector serotypes 1, 2, 5, 7, and 8 in mouse brain. Hum Gene Ther.

[5] Tervo DGR, Hwang B-Y, Viswanathan S, Gaj T, Lavzin M, Ritola KD (2016). A designer AAV variant permits efficient retrograde access to projection neurons. Neuron.

[6] Zingg B, Chou XL, Zhang ZG, Mesik L, Liang F, Tao HW (2017). AAV-mediated anterograde transsynaptic tagging: mapping corticocollicular input-defined neural pathways for defense behaviors. Neuron.

[7] Economo MN, Viswanathan S, Tasic B, Bas E, Winnubst J, Menon V (2018). Distinct descending motor cortex pathways and their roles in movement. Nature.

[8] Gründemann J, Bitterman Y, Lu T, Krabbe S, Grewe BF, Schnitzer MJ (2019). Amygdala ensembles encode behavioral states. Science.

[9] McCarty DM, Monahan PE, Samulski RJ (2001). Self-complementary recombinant adeno-associated virus (scAAV) vectors promote efficient transduction independently of DNA synthesis. Gene Ther.

[10] Grimm D, Lee JS, Wang L, Desai T, Akache B, Storm TA (2008). In vitro and in vivo gene therapy vector evolution via multispecies interbreeding and retargeting of adeno-associated viruses. J Virol.

[11] Zhong L, Li B, Jayandharan G, Mah CS, Govindasamy L, Agbandje-McKenna M (2008). Tyrosine-phosphorylation of AAV2 vectors and its consequences on viral intracellular trafficking and transgene expression. Virology.

[12] Zhong L, Li B, Mah CS, Govindasamy L, Agbandje-McKenna M, Cooper M (2008). Next generation of adeno-associated virus 2 vectors: point mutations in tyrosines lead to high-efficiency transduction at lower doses. Proc Natl Acad Sci U S A.

[13] Zhong L, Zhao W, Wu J, Li B, Zolotukhin S, Govindasamy L (2007). A dual role of EGFR protein tyrosine kinase signaling in ubiquitination of AAV2 capsids and viral second-strand DNA synthesis. Mol Ther.

[14] Douar AM, Poulard K, Stockholm D, Danos O (2001). Intracellular trafficking of adeno-associated virus vectors: routing to the late endosomal compartment and proteasome degradation. J Virol.

[15] Markusic DM, Herzog RW, Aslanidi GV, Hoffman BE, Li B, Li M (2010). High-efficiency transduction and correction of murine hemophilia B using AAV2 vectors devoid of multiple surface-exposed tyrosines. Mol Ther.

[16] Lipinski DM, Reid CA, Boye SL, Peterson JJ, Qi X, Boye SE (2015). Systemic vascular transduction by capsid mutant adeno-associated virus after intravenous injection. Hum Gene Ther.

[17] Petrs-Silva H, Dinculescu A, Li Q, Deng W-T, Pang J-J, Min S-H (2011). Novel properties of tyrosine-mutant AAV2 vectors in the mouse retina. Mol Ther.

[18] Ryals RC, Boye SL, Dinculescu A, Hauswirth WW, Boye SE (2011). Quantifying transduction efficiencies of unmodified and tyrosine capsid mutant AAV vectors in vitro using two ocular cell lines. Mol Vis.

[19] Kay CN, Ryals RC, Aslanidi GV, Min SH, Ruan Q, Sun J (2013). Targeting photoreceptors via intravitreal delivery using novel, capsid-mutated AAV vectors. PLoS ONE.

[20] Qiao C, Zhang W, Yuan Z, Shin J-H, Li J, Jayandharan GR (2010). Adeno-associated virus serotype 6 capsid tyrosine-to-phenylalanine mutations improve gene transfer to skeletal muscle. Hum Gene Ther.

[21] Dalkara D, Byrne LC, Lee T, Hoffmann NV, Schaffer DV, Flannery JG (2012). Enhanced gene delivery to the neonatal retina through systemic administration of tyrosine-mutated AAV9. Gene Ther.

[22] Uematsu A, Tan BZ, Ycu EA, Cuevas JS, Koivumaa J, Junyent F (2017). Modular organization of the brainstem noradrenaline system coordinates opposing learning states. Nat Neurosci.

[23] Hoshino C, Konno A, Hosoi N, Kaneko R, Mukai R, Nakai J (2021). GABAergic neuron-specific whole-brain transduction by AAV-PHP.B incorporated with a new GAD65 promoter. Mol Brain.

[24] Chan KY, Jang MJ, Yoo BB, Greenbaum A, Ravi N, Wu WL (2017). Engineered AAVs for efficient noninvasive gene delivery to the central and peripheral nervous systems. Nat Neurosci.

[25] Tanguy Y, Biferi MG, Besse A, Astord S, Cohen-Tannoudji M, Marais T (2015). Systemic AAVrh10 provides higher transgene expression than AAV9 in the brain and the spinal cord of neonatal mice. Front Mol Neurosci.

[26] Sun L, Tang Y, Yan K, Yu J, Zou Y, Xu W (2019). Differences in neurotropism and neurotoxicity among retrograde viral tracers. Mol Neurodegener.

[27] Summerford C, Samulski RJ (1998). Membrane-associated heparan sulfate proteoglycan is a receptor for adeno-associated virus type 2 virions. J Virol.

[28] Schmidt M, Govindasamy L, Afione S, Kaludov N, Agbandje-McKenna M, Chiorini JA (2008). Molecular characterization of the heparin-dependent transduction domain on the capsid of a novel adeno-associated virus isolate, AAV(VR-942). J Virol.

[29] Zhijian W, Edward M, Mavis AM, Jude SR (2006). α2,3 and α2,6 N-linked sialic acids facilitate efficient binding and transduction by adeno-associated virus types 1 and 6. J Virol..

[30] Kaludov N, Brown KE, Walters RW, Zabner J, Chiorini JA (2001). Adeno-associated virus serotype 4 (AAV4) and AAV5 both require sialic acid binding for hemagglutination and efficient transduction but differ in sialic acid linkage specificity. J Virol.

[31] Shen S, Bryant KD, Brown SM, Randell SH, Asokan A (2011). Terminal N-linked galactose is the primary receptor for adeno-associated virus 9. J Biol Chem.

[32] Bell CL, Gurda BL, Van Vliet K, Agbandje-McKenna M, Wilson JM (2012). Identification of the galactose binding domain of the adeno-associated virus serotype 9 capsid. J Virol.

[33] Summerford C, Samulski RJ (2016). AAVR: a multi-serotype receptor for AAV. Mol Ther.

[34] Li S-J, Vaughan A, Sturgill JF, Kepecs A (2018). A viral receptor complementation strategy to overcome CAV-2 tropism for efficient retrograde targeting of neurons. Neuron.

[35] Zingg B, Peng B, Huang J, Tao HW, Zhang LI (2020). Synaptic specificity and application of anterograde trans-synaptic aav for probing neural circuitry. J Neurosci.

