# Supplementary material for: The tyrosine capsid mutations on retrograde adeno-associated virus accelerates gene transduction efficiency
Source: Mol Brain. 2022 Aug 8;15:70. doi: 10.1186/s13041-022-00957-0 (PMC9358834; doi:10.1186/s13041-022-00957-0)
Supplement: Supplementary file 1 — Additional file 1: Fig. S1. The gene transduction efficiency of AAV2-retro in AAV-293 cell was significantly decreased by YF mutations. Fig. S2. The number of neurons labeled by AAV1 was significantly decreased in the V1-SC pathway. [file 13041_2022_957_MOESM1_ESM.docx]

**Additional Information**


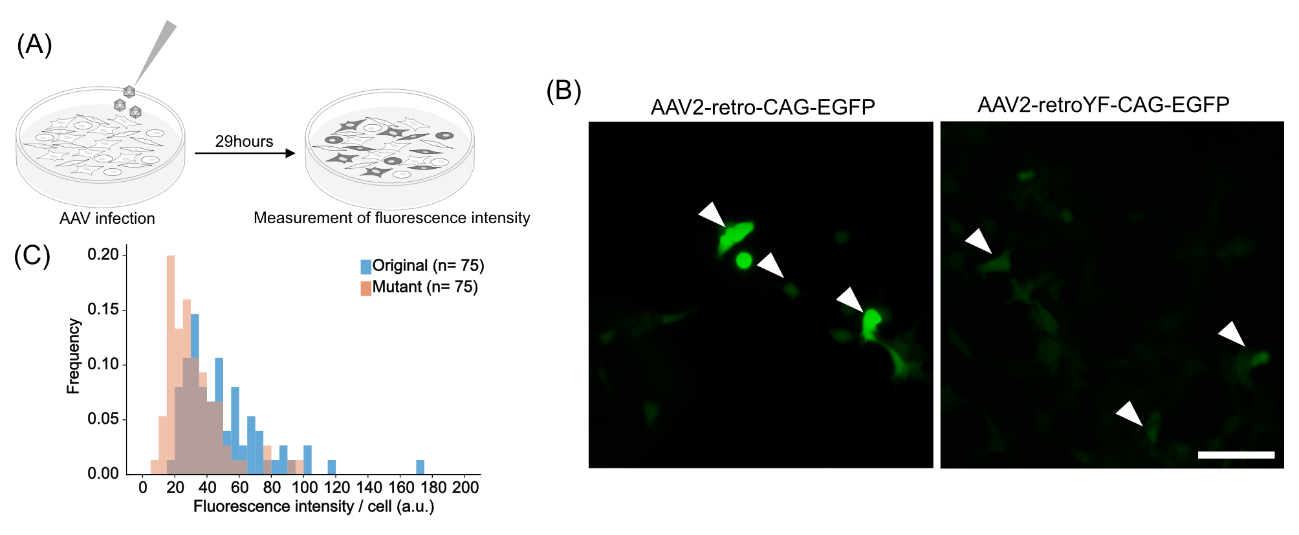


**Figure S1. The gene transduction efficiency of AAV2-retro in AAV-293 cell was significantly decreased by YF mutations.**

(A) An experimental procedure of *in vitro* AAV assay on AAV-293 cell. (B) Representative images of EGFP positive cells (green) of AAV2-retro-CAG-EGFP and AAV2-retroYF-CAG-EGFP. Scale bar, 100 μm. (C) The histogram of the fluorescent intensity of EGFP-positive cells. AAV2-retroYF significantly decreased EGFP intensity compared to AAV2-retro (n = 75 [original], n = 75[mutant], p < 0.001, Wilcoxon’s rank sum test).


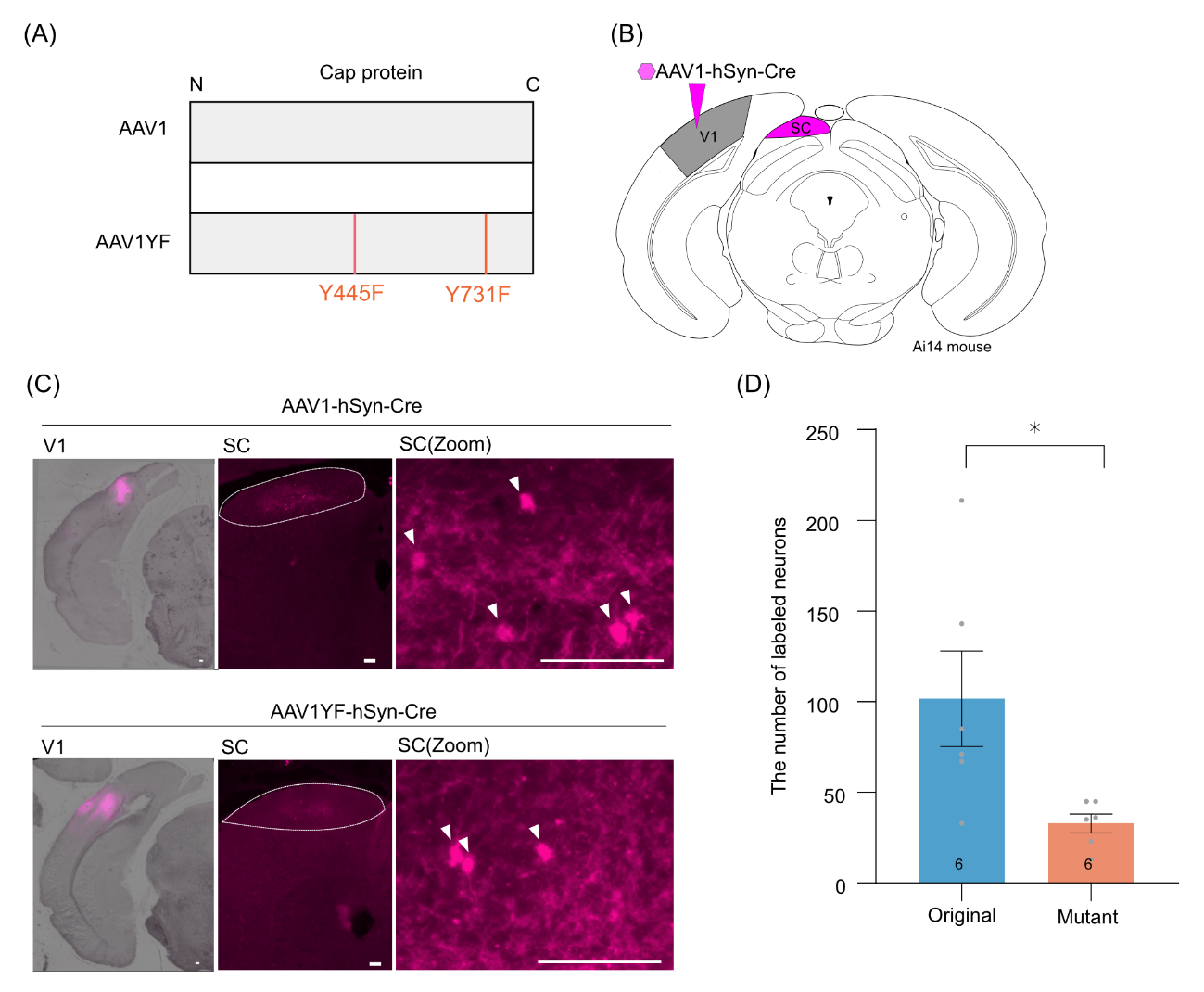


**Figure S2. The number of neurons labeled by AAV1 was significantly decreased in the V1-SC pathway**

(A) A schematic view of the YF mutations in AAV1YF. (B) A schematic of AAV1 injection site (V1) and imaging site (SC). AAV1 or AAV1YF was injected into the V1 of Ai14 mice. (C) Representative images of injection sites and tdTomato positive neurons (magenta) in the SC by AAV1-hSyn-Cre (top) and AAV1YF-hSyn-Cre (bottom). The white arrows indicate tdTomato-labeled neurons in the SC. Scale bar, 100 μm. (D) Quantification of the number of neurons labeled by tdTomato (mean ± sem; n = 6 [original], n = 6 [mutant], p = 0.046, Welch's two-tailed t-test, t = 2.565, df = 5.384).
